# Investigation on Furan Levels in Pressure-Cooked Foods

**DOI:** 10.1155/2013/904349

**Published:** 2013-01-14

**Authors:** Adriana P. Arisseto, Eduardo Vicente, Maria Cecília F. Toledo

**Affiliations:** Food Science and Quality Center, Institute of Food Technology, Avenida Brasil 2880, CP 139, 13070-178 Campinas, SP, Brazil

## Abstract

Furan is a food processing contaminant classified as possibly carcinogenic to humans. As the occurrence of furan has been reported in a variety of foods processed in sealed containers, the objective of this work was to investigate if the contaminant can be found in home-cooked foods prepared in a pressure cooker. For that, several foods including beans, soy beans, whole rice, beef, pork, potato, and cassava were pressure-cooked and analyzed for the furan content by gas chromatography coupled to mass spectrometry preceded by a headspace solid phase microextraction (HS-SPME-GC/MS). Furan was not found above the limit of quantification in the pressure-cooked samples. No furan has either been found in reheated samples after 24 hours under cold storage. Although levels up to 173 *μ*g/kg were already reported for commercial canned/jarred foods, it seems that the cooking in a pressure cooker may not represent a concern in relation to the occurrence of furan in foods.

## 1. Introduction

Furan (C_4_H_4_O) is a colorless flammable liquid with an ethereal odor, having a low molecular weight of 68 and a high volatility with the boiling point of 31°C [1]. Furan is classified as a possible human carcinogen (group 2B) by the International Agency for Research on Cancer in view of its hepatotoxicity, cytotoxicity, and carcinogenicity verified in experimental animals [2].

In 2004, American researchers demonstrated that furan can be formed during the thermal treatment of several foods, including canned/jarred products [3]. According to this study, levels up to 112 and 173 *μ*g/kg were found in jarred baby food and canned gravies, respectively. The occurrence of furan in canned and jarred foods has been attributed to the thermal treatment carried out in hermetically sealed containers which avoids losses of the contaminant by volatilization and permits its accumulation in the products [4]. 

In order to evaluate the processing parameters and mechanisms that lead to the furan formation in canned/jarred foods, pressure cooking conditions (in general 120°C for 20 min) have been used to simulate the sterilization step employed in a typical commercial canning operation in which the generation of the contaminant is supposed to occur [4–10]. These studies have confirmed that furan can be formed when model systems and retail foods are heated in sealed vials under the mentioned conditions. 

However, it should be emphasized that the pressure cooking is a common and widely used domestic practice which permits food to be cooked faster, as the pressure built up inside the cooker allows the liquid in the pot to rise to a temperature higher than 100°C. Considering that the furan formation under pressure cooking conditions has already been demonstrated in model systems and retail foods heated in sealed vials, it is important to evaluate if the contaminant can be found in home pressure-cooked foods. Moreover, the influence on furan levels of other practices commonly applied after the pressure cooking, such as an additional cooking without the lid and reheating after cold storage, was also investigated. 

## 2. Materials and Methods

### 2.1. Standards and Chemicals

Furan and [^2^H_4_] furan (furan-d_4_) were obtained from Sigma-Aldrich (Sigma-Aldrich Corp., St. Louis, MO, USA) at purity higher than 98%. Methanol was of HPLC-grade (Tedia Company Inc., Fairfield, OH, USA) and water was purified by reverse osmosis (Gehaka, São Paulo, SP, Brazil). Individual stock solutions of both standards at *ca* 2 mg/mL were prepared by dissolving in methanol. Intermediate and work solutions at *ca* 20 *μ*g/mL and 0.2 *μ*g/mL, respectively, were prepared in water.

### 2.2. Samples

Several foods were considered in the present study, including beans, soy beans, whole rice, beef, pork, potato, and cassava. The samples were obtained from the local market. With exception of beans, soy beans, and whole rice, the samples were cut in cubs of approximately 2 × 2 cm prior to cooking. Potato and cassava were previously peeled. 

### 2.3. Pressure Cooking

A domestic aluminium pressure cooker of 4.5 litres (Clock, Panex LTDA, São Bernardo do Campo, SP, Brazil) was used in the experiments. After the addition of 400 g of the fresh sample (prepared as mentioned above) and 1.5 L of water at room temperature, the cooker was immediately closed and the cooking was performed during 20 min after the first release of steam from the pressure valve. The samples were cooked and reheated directly in the water in order to reproduce the domestic preparation of soups and stews. 

Before opening, the pressure cooker was cooled under tap water for sufficient time to eliminate the pressure. Then, a portion of the cooked sample was collected as quickly as possible and properly stored until homogenization and analysis. The remained sample was subdivided in another two portions. The first one was kept in the pressure cooker and was cooked for additional 10 min without the lid. The second portion was stored under refrigeration during 24 hours and reheated in an open saucepan during 5 min after boiling. All samples were stored at 4°C for at least 4 hours before homogenization in order to avoid furan loss by volatilization.

### 2.4. Determination of Furan

The furan content was determined in the fresh, cooked, refrigerated, and reheated samples by using an in-house validated method based on gas chromatography coupled to mass spectrometry preceded by headspace solid phase microextraction (HS-SPME-GC/MS) according to Arisseto et al. [11]. Briefly, a portion of 1 g of homogeneous sample was weighed in a chilled 40 mL screw-cap glass vial fitted with silicone-PTFE septum containing a 15 mm × 5 mm PTFE-coated stir bar. Aliquots of 125 *μ*L of furan-d_4_ working standard solution 0.2 *μ*g/mL and 875 *μ*L of water were added and the vial was immediately closed. The SPME was carried out in a 75 *μ*m carboxen-polydimethylsiloxane (CAR-PDMS) fiber (Supelco, Bellefonte, PA, USA) at 25°C during 30 min, under a constant magnetic agitation rate of 1200 rpm, approximately. All samples were analysed in duplicate.

### 2.5. GC/MS Analysis

The analyses were performed on an HP 6890 gas chromatography equipped with an MSD 5973 mass spectrometer (Agilent Technologies, Palo Alto, CA, USA). Helium was used as the carrier gas at a constant flow rate of 0.7 mL/min. The Programmable Temperature Vaporizing (PTV) injector was operated in the splitless mode under the following temperature program: 40°C (held for 0.1 min), 700°C/min up to 230°C (held for 23 min). The split valve remained open for 0.7 min. The separation was performed on a 60 m × 0.25 mm, *d*_*f*_ 0.25 *μ*m HP-INNOWAX capillary column (Agilent Technologies) and the oven temperature program was 30°C (held for 0.1 min), 2°C/min up to 40°C (held for 3 min), and 12°C/min up to 200°C (held for 2 min). The mass spectrometer was operated in a positive electron impact ionization mode (+EI) with 70 eV of the electron energy. The quadrupole and the ionization source were maintained at 150 and 230°C, respectively. The selected ion monitoring (SIM) was used for the detection of furan and furan-d_4_, using *m/z* 68∗/39/69 for furan and *m/z* 72∗/42 for furan-d_4_ (∗quantifier ions). A dwell time of 100 ms was used for all the ions.

### 2.6. Identification and Quantification

The relative retention time (RRT) and the presence of characteristic ions were considered for identification of furan in the samples. For confirmatory purposes, an acceptable deviation of ±0.5% for RRT, ±10% for ionic relative abundance considering *m/z* 39/68, and ±50% for ionic relative abundance considering *m/z* 69/68 were used by comparing the sample with a standard solution [12]. The quantification of furan was carried out by extrapolation from a linear analytical curve, using the quantifier ions *m/z* 68 (furan) and *m/z* 72 (furan-d_4_, internal standard).

## 3. Results and Discussion

Although several studies have been published on furan levels in commercial products, few data is available for home-cooked foods. Fromberg et al. [13] evaluated the formation of furan during domestic cooking such as grilling, roasting, baking, frying, and cooking in saucepan and microwave. However, the presence of furan in home-cooked foods prepared in a pressure cooker has not been investigated so far.

**Figure 1:**
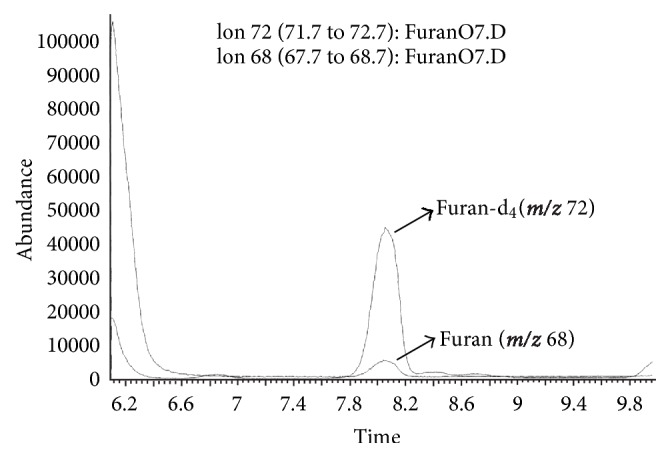
Ion chromatogram of a sample of cooked pork containing <2.4 *μ*g/kg (*m/z* 68 and 72: quantifier ions; carrier gas: helium; flow rate: 0.7 mL/min; Programmable Temperature Vaporizing (PTV) injector: 40°C (held for 0.1 min), 700°C/min up to 230°C (held for 23 min); mode: splitless; column: 60 m × 0.25 mm, *d*_*f*_ 0.25 *μ*m HP-INNOWAX; oven: 30°C (held for 0.1 min), 2°C/min up to 40°C (held for 3 min), 12°C/min up to 200°C (held for 2 min); mass spectrometer: positive electron impact ionization (70 eV); quadrupole temperature: 150°C; ionization source temperature: 230°C; dwell time: 100 ms).

Figure 1 illustrates a typical ion chromatogram of a pressure-cooked pork sample and the levels of furan obtained for the pressure-cooked foods selected in the present study are shown in Table 1. As expected, furan was not detected in the fresh samples. The results also indicate that the pressure cooking did not produce quantifiable levels of the contaminant for any of the evaluated samples.

Several furan derivatives were already identified in pressure-cooked meats. In a study on volatile constituents of pressure-cooked pork liver isolated by simultaneous steam distillation and continuous solvent extraction, Mussinan and Walradt [14] identified 23 furanic compounds. Other authors found out 3 furan derivatives between the most potent aromatic constituents of pressure-cooked hen meat [15]. However, the presence of the unsubstituted compound has not been reported in these studies, which is consistent with the results obtained in the present work.

Nevertheless, taking into account that the formation of furan has already been demonstrated in model systems and retail foods heated in sealed vials under pressure cooking conditions, it could be suggested that any furan possibly formed in the studied samples was lost with the steam that is released by the valve when a determined pressure is reached. This is in accordance with the observations reported by Hasnip et al. [4] that dried vegetables heated in open vials accumulated very little furan in comparison with sealed samples.

It should be noted that the possible release of furan to the air kitchen during the pressure cooking could contribute for an occupational exposure to the contaminant. This should be further investigated since it has already been demonstrated that furan was found in the air kitchen as a result of some procedures, such as the addition of water to a cafetiere, the frying of chipped potatoes in an open chip pan, and baking some foods in an oven [16].

**Figure 2:**
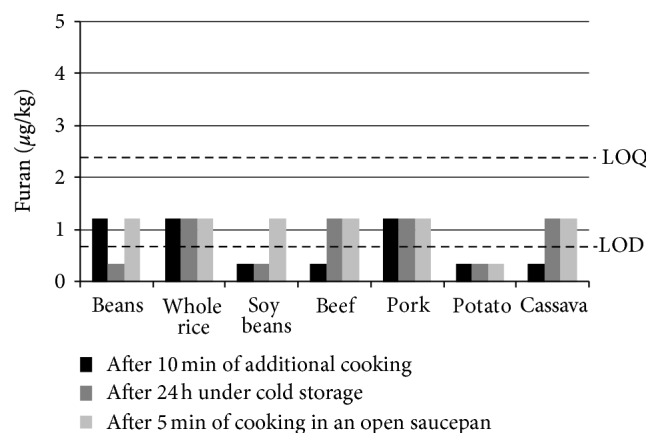
Furan levels obtained after reheating (for illustration purposes: levels below limit of detection (LOD) were considered as half of LOD (0.35 *μ*g/kg) and levels below limit of quantification (LOQ) were considered as half of LOQ (1.2 *μ*g/kg)).

Figure 2 illustrates the levels of furan obtained in the subsequent cooking experiments. It can be seen that no furan has also been found in quantifiable amounts in the samples cooked for additional 10 min without the lid as well as in the reheated samples after 24 hours under cold storage.

Data available in the literature on the furan content after the application of common warming procedures have shown conflicting results. Some authors reported furan losses of 29–85%, whereas others have found that furan persists during normal heating practices [4, 17, 18]. It has also been demonstrated that furan could even be formed at levels up to 29.2 *μ*g/kg during the reheating of baby foods containing potatoes after cold storage [19]. However, no significant furan loss or formation was observed in the samples investigated here.

It was very recently reported that additional amounts of furan can be formed from potential precursors such as 2-butenal and furfural during the thermal desorption of the fiber when using SPME-GC/MS [20]. Although this technique was applied in the present study, it seems that the general impact of the possible artifactual furan formation on the obtained results was negligible.

## 4. Conclusions

Furan has been reported in a variety of thermally processed foods, including canned/jarred commercial products as well as home-cooked foods obtained by grilling, roasting, baking, or frying. Although the conditions of pressure cooking can lead to furan formation in model systems and retail foods heated in sealed vials, as demonstrated by several studies, it seems that this procedure as applied at a domestic level may not represent a concern in relation to the occurrence of furan in home pressure-cooked foods. This is quite important considering that, to date, the research on furan has not been successful in identifying practical and consistently effective solutions for decreasing the levels of the contaminant in foods [21]. Also, as the interventions on furan reported in the scientific literature are mostly targeted at the level of the consumer rather than industrial production methods, the identification of domestic practices that could produce foods with reduced furan levels is highly desirable.

## Figures and Tables

**Table 1:** Furan levels in pressure-cooked foods.

Food	Furan (*μ*g/kg)	
	Raw	Cooked
Beans	nd	<2.4
Whole rice	nd	<2.4
Soy beans	nd	<2.4
Beef	nd	nd
Pork	nd	<2.4
Potato	nd	nd
Cassava	nd	nd

nd = values below the limit of detection (LOD = 0.7 *μ*g/kg).
